# Hypercholesterolemia triggers innate immune imbalance and transforms brain infarcts after ischemic stroke

**DOI:** 10.3389/fimmu.2024.1502346

**Published:** 2025-01-08

**Authors:** Ali Ata Tuz, Nils Hoerenbaum, Özgür Ulusoy, Adel Ahmadi, Alana Gerlach, Alexander Beer, Andreas Kraus, Anja Hasenberg, Nina Hagemann, Dirk M. Hermann, Matthias Gunzer, Vikramjeet Singh

**Affiliations:** ^1^ Institute for Experimental Immunology and Imaging, University Hospital Essen, University of Duisburg-Essen, Essen, Germany; ^2^ Department of Neurology, University Hospital Essen, University of Duisburg-Essen, Essen, Germany; ^3^ Department of Biospectroscopy, Leibniz-Institut für Analytische Wissenschaften - ISAS -e.V., Dortmund, Germany

**Keywords:** high-fat diet, hypercholesterolemia, stroke, neutrophils, inflammation

## Abstract

Post-stroke early activation of neutrophils contributes to intensive neuroinflammation and worsens disease outcomes. Other pre-existing patient conditions can modify the extent of their activation during disease, especially hypercholesterolemia. However, whether and how increased circulating cholesterol amounts can change neutrophil activation responses very early after stroke has not been studied. In this study, we investigated the effect of high-fat diet (HFD) induced hypercholesterolemia on neutrophil activation and stroke outcome. Mice were fed with HFD or normal diet (ND) for six weeks and then induced stroke by transient occlusion of the middle cerebral artery. The activation receptors on immune cells and plasma levels of cytokines were analyzed using flow cytometry. The amount of plasma neutrophil extracellular traps (NETs) was measured using citH3-DNA complex ELISA. We found that HFD-induced cholesterolemia increased the number of circulating and splenic neutrophils in stroke mice but reduced bone marrow neutrophils compared to sham controls. After stroke neutrophils in HFD mice expressed higher levels of activation markers Ly6G and PSGL-1 (CD162) compared to ND mice. In addition, stroke led to an increased expression of the activation markers Ly6C and CD68 on monocyte/macrophages (MΦ) in HFD mice but not in ND mice. Compared to ND, HFD increased plasma levels of the proinflammatory cytokines TNF-α, IL-6, IL-23, and MCP-1 in stroke mice. Remarkably, HFD mice showed higher amounts of circulating NETs, brain-infiltrated neutrophils, and larger infarcts after stroke compared to ND mice. The existence of hypercholesterolemia with a stroke can trigger a stronger activation of neutrophils and MΦ, causing deteriorating disease outcomes.

## Introduction

Ischemic stroke is a major disorder with high morbidity and mortality in affected patients. Therapeutic interventions to treat stroke are still limited to thrombolysis using tissue plasminogen activators or mechanical thrombectomy (1, 2). Within a few hours after stroke, innate immune cells in systemic lymphoid tissues are activated and infiltrate the injured brain. Specifically, activated neutrophils and monocytes/macrophages (MΦ) can enter the brain parenchyma as early as six hours after the onset of the first symptoms (2). They can release a plethora of neurotoxic molecules such as free radicals, matrix metalloproteases, cytokines, and neutrophil extracellular traps (NETs) which promote neurodegeneration (3–5). A higher number of circulating neutrophils are present in stroke patients and associated with deteriorated disease outcomes (6). The majority of stroke patients suffer from other comorbidities such as obesity, hypercholesterolemia, and diabetes (7, 8) and higher circulating amounts of cholesterol have been linked to the increased risk of stroke and poor outcomes (9, 10). Previously, we have shown that ApoE^−/−^mice fed with a high-fat diet (HFD) develop a more severe breakdown of the blood-brain barrier (BBB), increased brain inflammation, and edema within 24 h of ischemia-reperfusion injury (11). Whether HFD-induced hypercholesterolemia influences very early (3 h post-stroke) activation of neutrophils and MΦ in wild-type mice has not been studied until now.

For this investigation, wild-type mice were fed with HFD or normal diet (ND) for six weeks before stroke. We found that mice fed on HFD showed accelerated neutrophil and MΦ activation and increased amounts of circulating proinflammatory cytokines and neutrophil extracellular traps (NETs) after stroke compared to ND mice. Moreover, HFD mice after stroke suffered increased brain infarcts and neutrophil accumulation compared to ND mice. These findings may help to consider neutrophil activation as a prognostic marker in combination with clinical diagnosis to estimate stroke severity and therapeutic targets to improve the outcomes, especially in hyperlipidemic patients.

## Materials and methods

### Animals

All animal experiments were performed following ethical guidelines and were approved by the local authorities (G1713/18) of the Landesamt für Natur, Umwelt und Verbraucherschutz Nordrhein-Westfalen, Recklinghausen, Germany). Four-week-old male C57BL/6JHsd wild-type mice were received from Envigo, Netherlands. Mice were randomly divided into two groups and received a normal laboratory diet (Cat. 1329 FORTI, Ssnif) or a high-fat Western-style diet (Cat. E15721-347, Ssnif) for 6 weeks. Afterward, mice underwent sham or stroke surgery (transient middle cerebral artery occlusion, tMCAO) and were sacrificed three hours after the operation. The animals had ad libitum access to food and water. All experiments were performed and reported according to ARRIVE guidelines (12).

### Experimental mouse model of stroke

Brain injury was induced by tMCAO in C57BL/6JHsd mice (aged 11 weeks) anesthetized with 1.5% isoflurane in 100% oxygen. Mice were injected with the analgesic buprenorphine (0.1 mg/kg body weight, s.c.) 30 min before the surgery. An eye ointment (Bepanthen) was applied to avoid any harm to mouse eyes during the surgical procedures. A small incision was made between the ear and the eye to expose the temporal bone and a laser Doppler flow probe was attached to the skull above the core of the middle cerebral artery (MCA) territory. Mice were then placed in a supine position on a feedback-controlled heat pad and the midline neck region was exposed with a small incision. The common carotid artery (CCA) and left external carotid arteries were identified and ligated. A 2 mm silicon-coated filament (Cat. 702234PK5Re; Doccol) was inserted into the internal carotid artery to occlude the MCA. Brain ischemia was validated by a stable reduction of blood flow to ≤ 20% of baseline that was observed on the laser Doppler flow device. After 60 min of occlusion, the filament was removed for the reestablishment of blood flow. Mice were then injected with the anti-inflammatory drug carprofen (4-5 mg/kg body weight, s.c.), wounds were carefully sutured and mice returned to their cages with free access to food and water. Sham-operated mice underwent the same surgical protocol except the filament was inserted into the CCA and immediately removed. The exclusion criteria for experimental mice were as follows: inadequate ischemia (reduction of blood flow does not reach the ≤ 20% of baseline threshold), weight loss >20% of baseline body weight during the study, and spontaneous animal death.

### Flow cytometry analysis

Mice were deeply anesthetized with Ketamine/Xylazine (100 mg/kg/10 mg/kg, i.p.), and blood was collected via cardiac puncture and added to EDTA-containing tubes. Mice were then perfused with PBS and spleen and BM were collected in cold PBS. Single-cell suspensions were prepared by mincing the spleens in PBS and filtering through 70 µm cell filters. Spleen and blood samples were treated with erythrocytes lysis buffer and washed in PBS. The tibia was harvested through an incision a few millimeters below the knee joint and removed from muscle tissue. Both ends of the tibia were cut with sharp sterile scissors, and the bone marrow was flushed out with a 30-gauge needle and filtered through 30 µm cell filters. Single-cell suspensions were kept on ice and total cells per organ were calculated using an automated cell counter (Nexcelom Bioscience). From each sample, 5x10^5^ cells were stained with fluorochrome-conjugated antibodies against different cell surface markers. Antibodies used were CD45 (30-F11), CD11b (M1/70), Ly6G (1A8), CD68 (FA-11), Ly6C (HK1.4), CD62L (MEL-14) and CD162 (4RA10) were purchased from Biolegend, Germany. Cells were washed with cold PBS and suspended in a flow cytometry buffer. Samples were analyzed using MacsQuant Analyzer 16 (Miltenyi) and FlowJo software (BD Biosciences).

### Quantification of mice plasma NETs

The EDTA blood was centrifuged at 3000 g and the supernatant was collected for a second centrifuge at 8000 g for 10 minutes. The quantification of NETs was performed as previously described capture ELISA, which is based on citrullinated histone H3 associated with DNA (13). Antihistone H3 antibody (5 µg/ml; ab5103, Abcam) was coated overnight at 4°C onto 96-well plates followed by 5% BSA blocking for 2 h. Wells were three times washed with 300 µl washing buffer followed by the addition of 50 µl plasma and 80 µl incubation buffer (including peroxidase-labeled anti-DNA antibody) for 2 h at 300 rpm (Cell Death ELISAPLUS, Cat. 11774425001, Roche). Then, the wells were washed three times with 300 µl washing buffer and 100 µl peroxidase substrate was added to the wells for 30 min in the dark. Afterward, 100 µl ABTS peroxidase stop solution was added to the wells, and absorbance was measured at 405 nm and was subtracted by absorbance at 490 nm (Abs 405 nm− Abs 490 nm). The absorbance values were considered in direct proportion to the amounts of soluble NETs and were presented as a relative increase to control.

### Quantification of plasma lipids

The EDTA blood was centrifuged at 3000 g and the supernatant was collected for a second centrifuge at 8000 g for 10 minutes. Finally, plasma samples from HFD and ND-treated stroke mice were analyzed for total cholesterol levels using a bioanalyzer (ADVIA^®^ 2400; Siemens, Erlangen, Germany).

### Quantification of plasma cytokines

Plasma cytokines were measured using a bead-based Legendplex immunoassay according to the manufacturer’s instructions (Cat. 740622, Biolegend). Briefly, 12.5 µl of mouse plasma was diluted with an assay buffer. All diluted samples, standards, and controls were added to the V-shaped 96-well plate. Supplied antibodies-specific beads were vortexed and added to samples in the wells and incubated for 2 h at room temperature with continuous shaking. Afterward, samples and technical controls were centrifuged and washed three times and biotinylated detection antibodies were added to the samples for 1 h at room temperature. Without washing, streptavidin-phycoerythrin is subsequently added, which will bind to the biotinylated detection antibodies providing fluorescent signal intensities in proportion to the amount of bound analytes. Samples were washed and acquired on a flow cytometer (BD FACSAria). Different cytokine amounts were quantified against the acquired standard curve using LEGENDplex software.

### Quantification of mice brain infarct volumes

Mice were sacrificed and perfused with 20 ml PBS followed by perfusion with 20 ml 4% PFA. Brains were carefully removed from the skull bone and transferred to 4% PFA for 12 h at 4°C. The next day, PFA was replaced by 30% sucrose solution and samples were incubated for 2 days at 4°C. Samples were tap-dried with tissue paper and quickly frozen in cold isopentane. Brains were transferred to −80°C until further processing. Cryosections of 20 μm thickness were prepared at 500 µm intervals from the neocortex and stained with cresyl violet (Cat. C5042, Sigma). All sections were scanned using 600 dpi and analyzed using the ImageJ software (NIH). Using a scale of 23.62 pixels/mm, the area of non-stained infarct tissue was measured and integrated into the total brain. An edema correction for brain infarct volume was performed using the following formula: (ischemic area) = (direct lesion volume) − [(ipsilateral hemisphere) − (contralateral hemisphere)]. The lesion volume per hemisphere was presented as mm^3^.

### Staining and analysis of whole brains using LSFM

Mice were deeply anesthetized and intravenously injected with fluorochrome-conjugated 5 µg anti-Ly6G (1A8, Cat.127648, Biolegend) and 7.5 µg anti-CD31 (MEC13.3, Cat.102528, Biolegend) antibodies. After 30 minutes, mice were sacrificed and transcardially perfused with 20 ml PBS and 20 ml of 4% PFA. Samples were incubated in 4% PFA at 4°C for 12 h. The next day, brains were washed in PBS and dehydrated with serial dilutions of tetrahydrofuran (THF) 30-60-80-100% 12 h each at RT with constant shaking at 70 rpm. The last 100% THF incubation was repeated twice and refractory index matching was achieved by immersion of the samples in ethyl cinnamate (ECI, Cat.112372, Sigma). Optically transparent samples were imaged via LSFM (Ultramicroscope BLAZE, Miltenyi Biotec) with a 40% light sheet width and a sheet thickness of 4 µm. The images were acquired with a zoom factor of 6.64x (Objective LVBT 4x, zoom 1.66x) and a step size of 2 µm for a total volume of 4.5 mm^3^ per ipsilateral hemisphere. The acquired 3D images were converted via the Imaris file converter and processed using Imaris software version 10.1.0. (Bitplane, Switzerland). Ly6G^+^ surfaces were considered as neutrophil-positive areas. Imaris surface function was used to create the respective areas and neutrophil surfaces per mm^3^ of brain volumes were quantified and presented.

### Statistical analysis

Data were analyzed using GraphPad Prism version 9.0. All datasets were tested for normal distribution using the Shapiro–Wilk normality test. The comparisons between the two groups were analyzed using the two-tailed Mann–Whitney U test. For statistics between more than two groups, ordinary one-way ANOVA with Tukey’s multiple comparisons tests was used for normally distributed data. The Kruskal-Wallis test was performed for comparisons of more than two groups followed by the Dunn’s multiple comparisons test for not normally distributed data. Differences with p-values ≤ 0.05 were considered to be statistically significant.

## Results

### HFD impacts neutrophil dynamics across systemic lymphoid tissues after stroke

**Figure 1:**
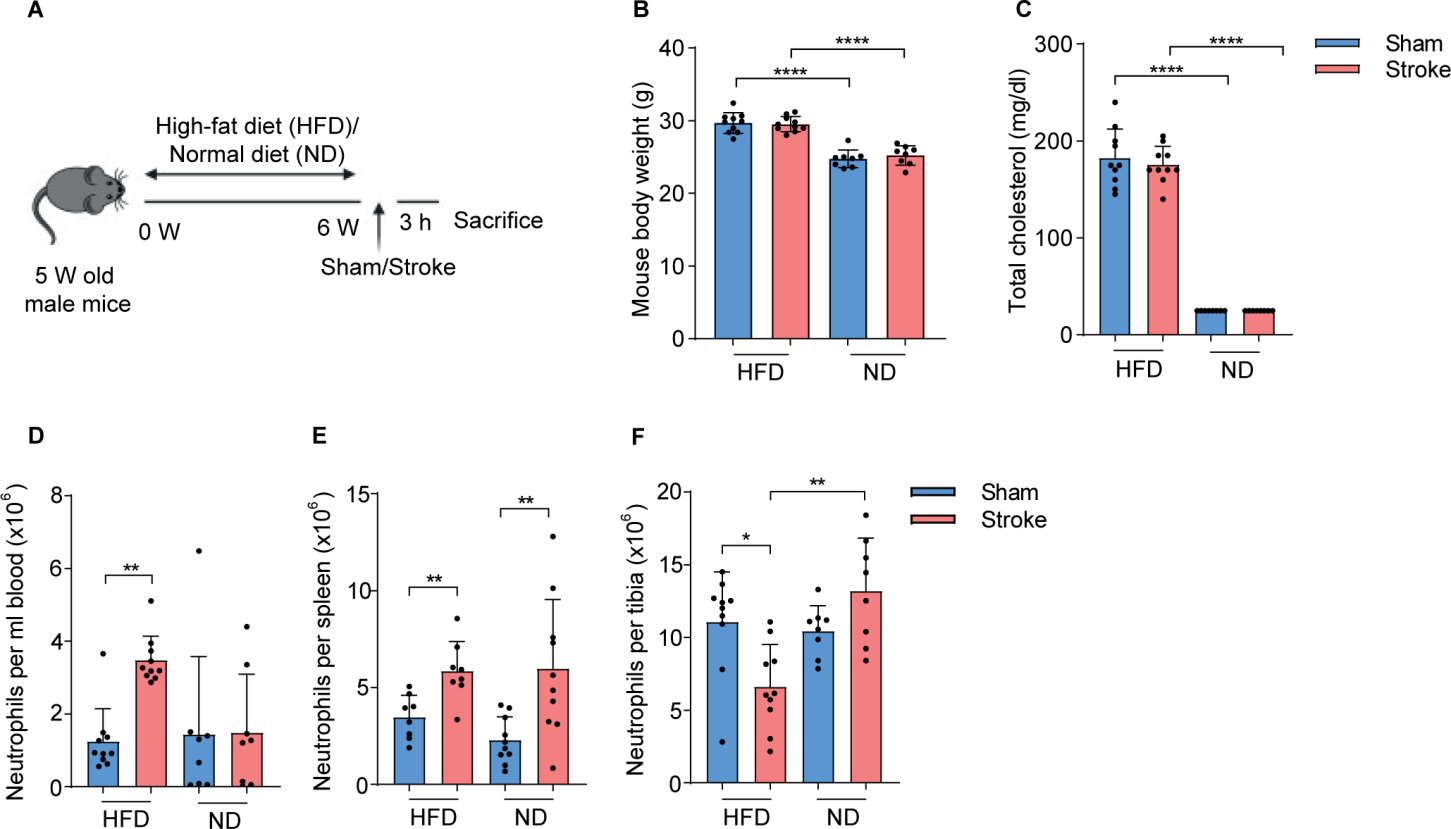
HFD increases circulating cholesterol levels and neutrophil numbers in stroke mice. **(A)** Experimental paradigm shows the treatment of mice with HFD or ND and the induction of stroke or sham operation. **(B)** Total body weight of sham-operated or stroke mice fed on HFD or ND. **(C)** Plasma amounts of cholesterol in HFD and ND mice 3 h after stroke or sham-operation. **(D)** The number of neutrophils in the blood, **(E)** spleen, and **(F)** tibial bone marrow of HFD and ND mice 3 h after stroke or sham-operation. Data are mean ± s.d., statistical analyses were performed by the Kruskal-Wallis test for multiple comparisons. *P<0.05, **P<0.01, ****P<0.0001, N=8-10 mice per group. HFD, high fat diet; ND, normal diet.

To investigate if the higher plasma cholesterol levels were linked to early innate immune activation after stroke, we induced hypercholesterolemia in mice with HFD for six weeks before the induction of stroke or sham operation (**Figure 1A**). As expected, HFD mice showed significantly increased body weight and higher plasma cholesterol amounts compared to ND mice and these levels remained unchanged at 3 h post-stroke compared to sham controls (**Figures 1B, C**). In addition, HFD mice had a higher liver-body weight ratio compared to ND mice (**Supplementary Figure 1A**).

Next, we analyzed the dynamics of neutrophils in systemic lymphoid tissues in HFD and ND mice after the induction of stroke or sham operation using flow cytometry (**Supplementary Figure 1B**). Our data showed that HFD increased the number of neutrophils in blood and spleen but their numbers were reduced in tibial bone marrow after stroke compared to sham controls (**Figures 1D–F**). In ND mice, the total number of neutrophils was only increased in the spleen after stroke compared to sham and bone marrow remained unaffected. In addition, a strong increase in the frequencies of blood and splenic neutrophils along with their decreased frequencies in tibial bone marrow after stroke in HFD mice indicated an accelerated neutrophil release to the circulation (**Supplementary Figures 1C–E**). However, stroke only increases the frequencies of splenic neutrophils compared to sham-operated ND mice. These data suggest that HFD-induced hypercholesteremia promotes post-stroke neutrophil activation and possibly increases their release from bone marrow.

### HFD changes the expression profile of neutrophil and MΦ activation receptors after stroke

**Figure 2:**
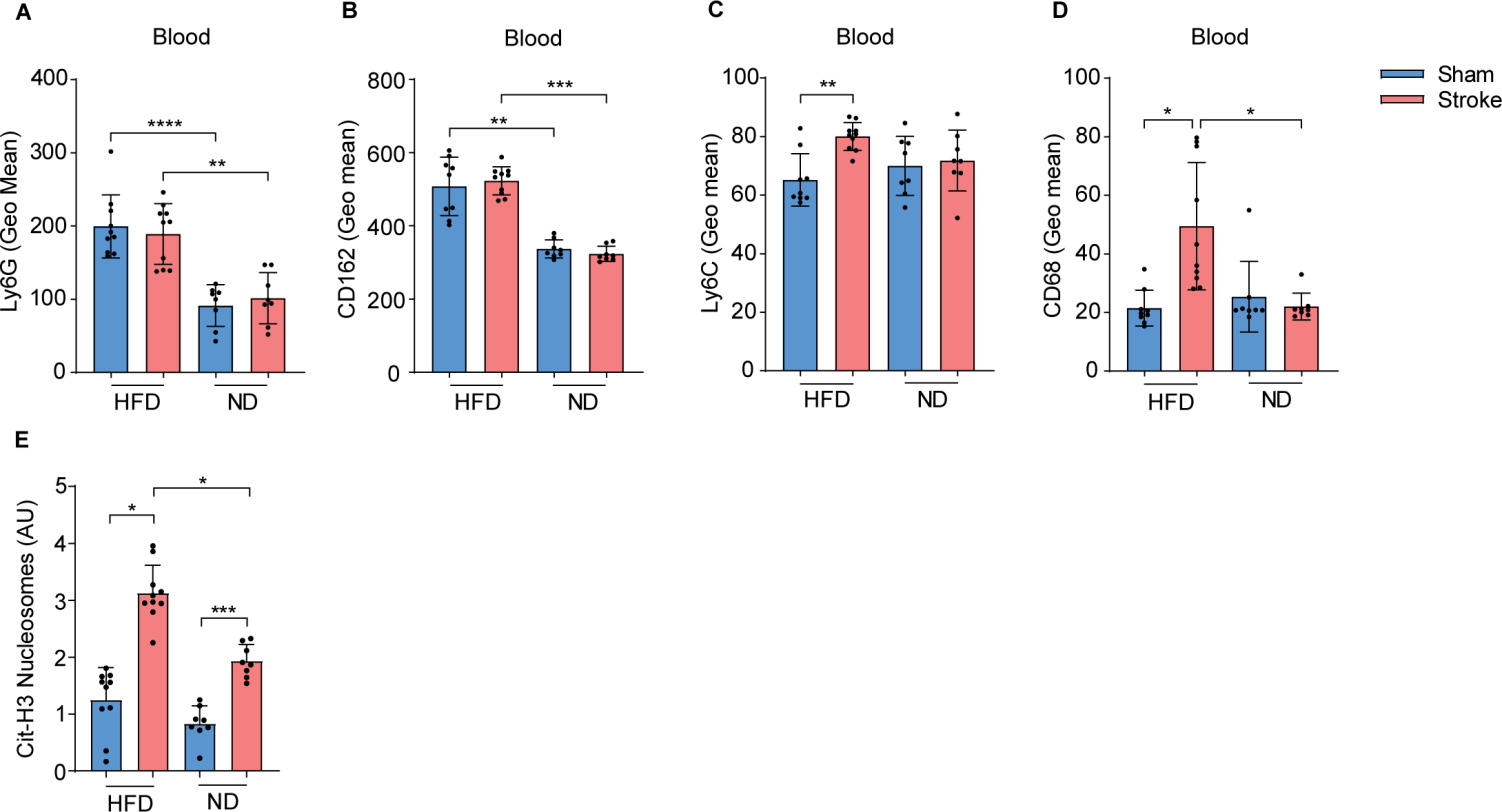
HFD triggers blood neutrophil and monocyte activation in stroke mice. **(A)** Mean fluorescence intensity (MFI) of Ly6G and **(B)** CD162 on blood neutrophils in HFD and ND mice after 3 h of stroke or sham operation. **(C)** MFI of Ly6C and **(D)** CD68 on MΦ in HFD and ND mice after stroke or sham operation. **(E)** The plasma amounts of cit-H3 nucleosomes in HFD and ND mice after stroke or sham operation. Data are mean ± s.d., statistical analyses were performed by the Kruskal-Wallis test for multiple comparisons. *P<0.05, **P<0.01, ***P<0.001, ****P<0.0001. N=8-9 mice per group. HFD, high fat diet; ND, normal diet; MΦ, monocyte/macrophage; Cit-H3, citrullinated histone H3.

To determine the activation pattern of neutrophils in HFD and ND mice, we compared the expression of different surface activation and adhesion receptors in blood, spleen, and tibial bone marrow after the induction of stroke or sham operation. The results showed that circulating neutrophils in HFD mice were characterized by a significantly higher expression of Ly6G (**Figure 2A**) and adhesion receptor CD162 (**Figure 2B**) compared to ND mice. However, stroke induction only increased the expression of CD162 and not Ly6G in splenic neutrophils in HFD mice compared to sham controls (**Supplementary Figures 1F, G**). In addition, we analyzed the activation pattern of MΦ after stroke in the blood and spleen of HFD and ND mice. Interestingly, our data showed that stroke in HFD mice upregulates the expression of a surrogate marker of activation Ly6C and the co-stimulatory receptor CD68 on blood MΦ (**Figures 2C, D**). However, no increase in the expression of these receptors was observed in ND mice after stroke compared to the sham group. Similarly, stroke increased the expression of CD68 but not Ly6C on splenic MΦ in HFD mice (**Supplementary Figures 1H, I**). Moreover, we found higher levels of plasma citH3-DNA nucleosomes as a measure of NET−formation in stroke mice with HFD compared to ND mice (**Figure 2E**). These results highlight the modulated responses of neutrophils and MΦ in systemic lymphoid tissues in response to HFD-induced hypercholesteremia and the induction of stroke.

### HFD triggers post-stroke proinflammatory cytokines release, neutrophil brain invasion, and worsens disease outcome

**Figure 3:**
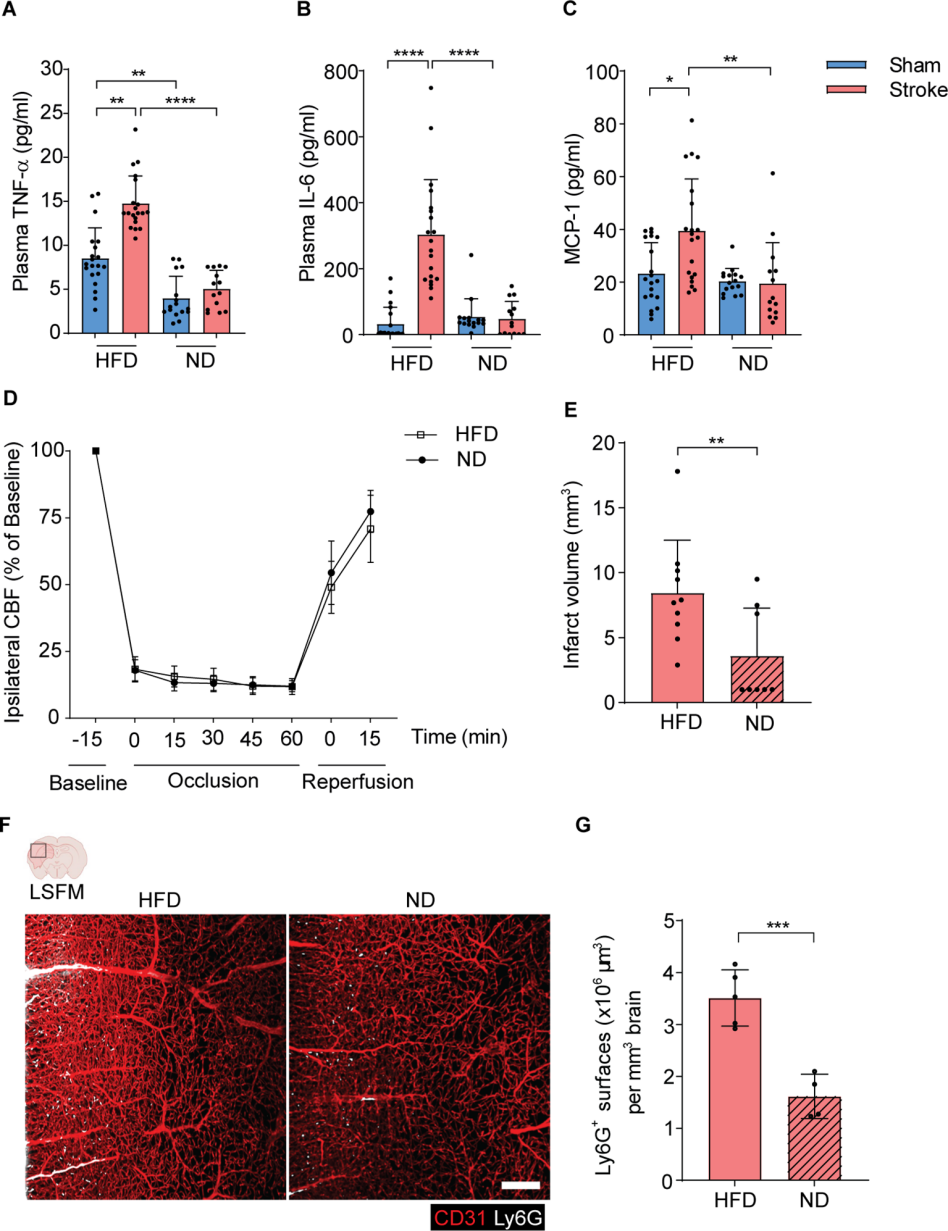
HFD triggers the release of inflammatory cytokines and increases brain infarct volumes after stroke. **(A–C)**. The amounts of plasma TNF-α, IL-6, and MCP-1 in HFD and ND-treated mice after 3 h of stroke or sham operation. **(D)**. The CBF was measured by Doppler recordings during ischemia-reperfusion injury in HFD and ND mice. **(E)**. The quantitative analysis of infarct volume using cresyl violet stained histological brain sections in HFD and ND mice. **(F)**. 3D rendering of ipsilateral brain hemispheres after stroke in HFD and ND mice showing higher frequencies of infiltrated neutrophils in HFD mice. Red=CD31, White=Ly6G, scale bar=200 μm. **(G)**. The total surface volumes of Ly6G in the ischemic hemispheres of HFD and ND mice. Data are mean ± s.d., statistical analyses were performed by the Kruskal-Wallis test for multiple comparisons or the Mann-Whitney U test for two-group comparisons. *P<0.05, **P<0.01, ***P<0.001, ****P<0.0001. N=8-9 mice per group. HFD, high-fat diet; ND, normal diet; CBF, cerebral blood flow; LSFM, light-sheet fluorescence microscopy.

Next, we analyzed the circulating amounts of proinflammatory cytokines in HFD and ND mice after stroke. Interestingly, stroke mice with HFD showed a significant upregulation of plasma TNF-α, IL-6 and MCP-1 amounts compared to sham controls within 3 h after stroke onset (**Figures 3A–C**). However, this rapid increase was not observed in stroke mice with ND. HFD sham mice have also increased plasma levels of TNF-α compared to ND mice. Furthermore, we found that stroke mice with HFD or ND show similar reduction and reperfusion of cerebral blood flow during laser Doppler flow (LDF) recordings (**Figure 3D**). In both groups, LDF reproducibly decreased to ~15-20% of baseline values during MCAO, followed by the reconstitution to ~75% within 15 minutes after reperfusion. Histological analysis of brain tissues of stroke mice showed large infarct volumes in HFD mice compared to ND controls after 3 h of ischemia-reperfusion injury (**Figure 3E**).

To further elucidate the underlying pathophysiology of increased brain infarcts in HFD mice after stroke, we implemented our three-dimensional light-sheet fluorescence microscopy (3D-LSFM) approach to analyze the extent of activated neutrophils migration to ischemic hemispheres (**Supplementary Figure J**). Our imaging data showed an augmented accumulation of neutrophils in the ipsilateral brain microvasculature and parenchymal tissue in HFD mice compared to ND mice (**Figure 3F**). Furthermore, the automated quantitative surface volume analysis showed a significant increase in Ly6G surface volumes in the ischemic brain hemispheres of HFD mice compared to ND mice (**Figure 3G**).

## Discussion

In this present study, we revealed that HFD triggers immune cell activation and contributes to increased hyperacute systemic inflammation after experimental stroke. Using multi-color flow cytometry analysis of neutrophils and MΦ in different lymphoid tissues, we found a substantial impact of HFD on the expression of cell activation receptors after stroke. HFD also led to higher amounts of proinflammatory cytokines in plasma and larger brain infarcts accompanied by increased brain infiltration of neutrophils within 3 h of ischemia-reperfusion injury. These findings have implications for utilizing neutrophil activation in combination with clinical investigations as a predictive marker for worse stroke outcomes in stroke patients with hypocholesteremia.

Stroke patients often have high levels of circulating cholesterol which is associated with poor outcomes and high mortality (11, 14). Previous clinical trials focusing on reducing lipids using statins have shown beneficial effects in stroke patients (15). A large number of clinical trials have demonstrated that a high neutrophil-to-lymphocyte ratio (NLR) is a prognostic marker of stroke severity, especially within 24 h of hospital admissions (16, 17). However, whether hypercholesteremia can modulate neutrophil and MΦ activation in an early therapeutic time window of 3 h after stroke and thereby further enhance inflammatory brain damage after stroke is not fully clear. We show that mice fed on HFD showed higher cholesterol levels and body weights. This led to increased numbers of circulating and splenic neutrophils which is in line with previous findings demonstrating the effect of hyperlipidemia on enhanced myelopoiesis and monocytosis in a genetic mouse model of obesity (18). Similarly, a recent study presented increased blood neutrophil numbers and their activation responses in ApoE^−/−^ mouse model of atherosclerosis (19).

Obesity is linked with low-grade inflammation mediated via constitutive activation of innate immune cells. For example, a higher amount of TNF-α is present in the adipose tissue of obese mice (20). We found higher amounts of inflammatory cytokines like TNF- α and IL-6 and chemokine MCP-1 in the plasma of HFD compared to ND stroke mice. Moreover, neutrophils and MΦ in HFD stroke mice also showed a rapid and stronger upregulation of activation, adhesion, and emigration receptors compared to ND mice. Previous studies have shown that high cholesterol and oxidized lipids can promote NET formation by the activation of inflammasome pathways in neutrophils (21). In addition, obesity can promote monocyte activation and in turn, upregulate NET release (22). We and others have also revealed a rapid and substantial effect of stroke on the release of toxic NETs in human stroke patients and after experimental stroke (23, 24). Our findings extend these observations with hypercholesterolemia and demonstrate its accelerating effect on the release of NETs very rapidly after stroke. Stroke mice with HFD have increased brain infarcts, consistent with previous findings in a rat model of ischemia-reperfusion injury (25). The worsening of stroke outcomes in mice can be related to an over-activation of neutrophils and their increased invasion of the ischemic brain observed in this study. However, a direct influence of HFD-induced metabolic disturbances on brain inflammation cannot be ruled out (26).

In summary, our study signifies the contribution of HFD-induced metabolic alterations in the early activation of neutrophils and MΦ following ischemic stroke. This information might provide a useful link to hypercholesteremia-driven neutrophil activation and test early treatment strategies to reduce immune activation and improve stroke outcomes.

## Conclusion

High-fat diet-induced hypercholesteremia is accelerating globally. Our study shows its contribution to enhanced systemic inflammation and prompt brain infarct growth after stroke.

## Data Availability

The original contributions presented in the study are included in the article/**Supplementary Material**. Further inquiries can be directed to the corresponding author.
