# Supplementary material for: Hypercholesterolemia triggers innate immune imbalance and transforms brain infarcts after ischemic stroke
Source: Front Immunol. 2025 Jan 8;15:1502346. doi: 10.3389/fimmu.2024.1502346 (PMC11750678; doi:10.3389/fimmu.2024.1502346)
Supplement: Supplementary file 1 [file Image1.pdf]

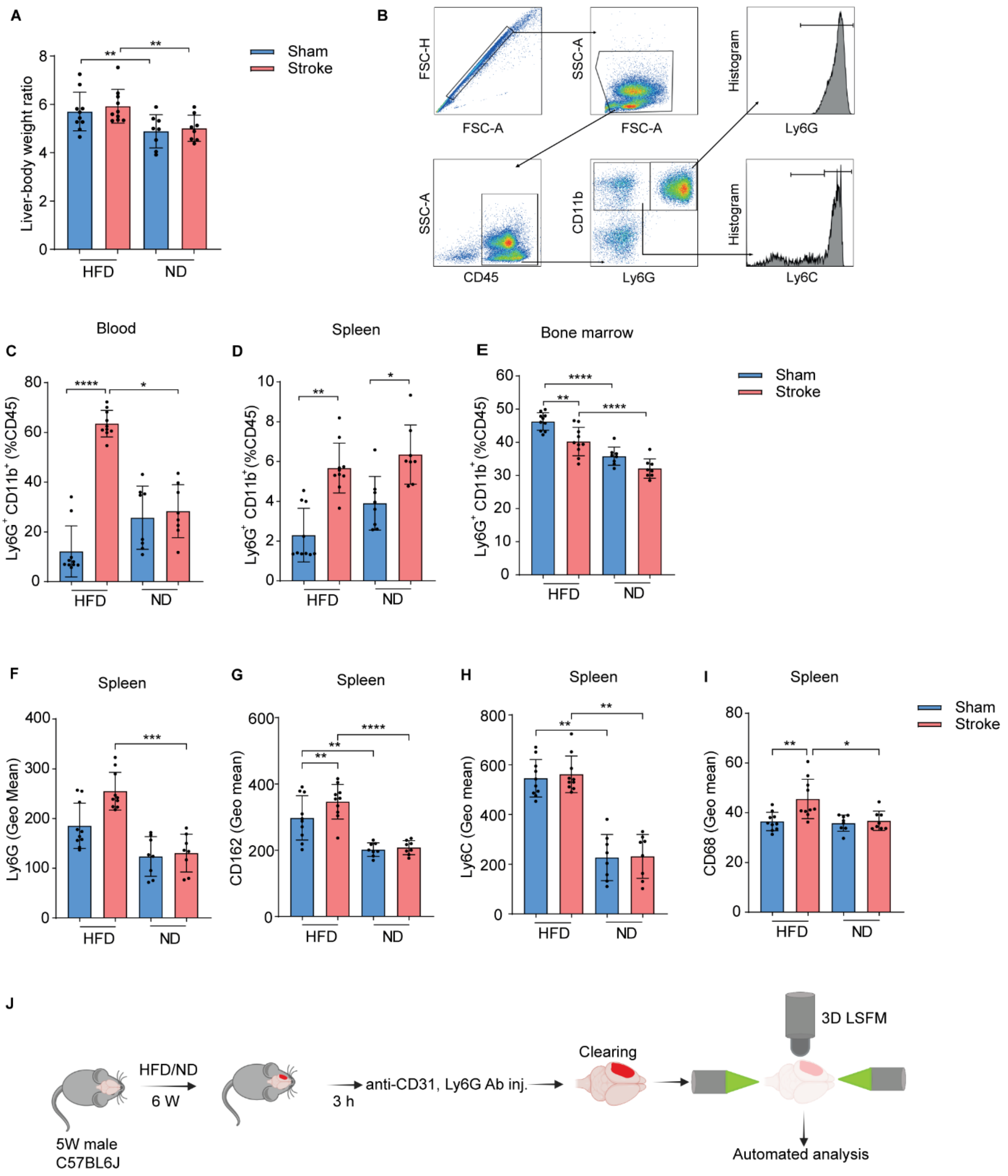

Supplementary Figure 1. HFD alters the activation of innate immune cells after stroke. A. Liver-body weight ratio of HFD or ND mice after 3 h of stroke or sham operation. **B.** Representative gating strategy for analyzing neutrophils and MΦ using multi-color flow cytometry. **C-E.** The frequencies of Ly6G$^+$CD11b$^+$ neutrophils in blood, spleen, and tibial bone marrow in HFD and ND mice after stroke or sham operation. **F.** Mean fluorescence intensity (MFI) of Ly6G and **G.** CD162 on splenic neutrophils in HFD and ND mice after stroke or sham operation. **H.** MFI of Ly6C and **I.** CD68 on MΦ in HFD and ND mice after stroke or sham operation. **J.** The schematic of 3D-LSFM brain imaging of HFD and ND stroke mice. Data are mean ±s.d., the Kruskal-Wallis test or ordinary one-way ANOVA performed statistical analyses for multiple comparisons. *P<0.05, **P<0.01, ***P<0.001, **** P<0.0001. N=8-9 mice per group. HFD= high-fat diet, ND= normal diet, MΦ=monocyte/macrophage, LSFM=light-sheet fluorescence microscopy.
